# Genotype-first analysis of a generally healthy population cohort supports genetic testing for diagnosis of hereditary angioedema of unknown cause

**DOI:** 10.1186/s13223-019-0346-1

**Published:** 2019-05-16

**Authors:** Dale L. Bodian, Thierry Vilboux, Natalie S. Hauser

**Affiliations:** Inova Translational Medicine Institute, Inova Health System, Falls Church, VA USA

**Keywords:** Nextgen sequencing, Clinical sequencing, Hereditary angioedema, Angioedema, Plasminogen, PLG, Differential diagnosis, Personalized medicine, Genetic testing, Diagnostic yield

## Abstract

**Background:**

Hereditary angioedema (HAE) is a potentially life-threatening group of conditions that is often underdiagnosed or misdiagnosed. As HAE is typically diagnosed by detecting C1 inhibitor deficiency, there is a critical need for methods that can identify affected individuals with normal C1 inhibitor. The recent discovery of associations between PLG K330E and ANGPT1 A119S and HAE of unknown genetic cause (HAE-U), has raised the possibility that genetic evaluation could be used to diagnose HAE-U in patients with unexplained angioedema or non-confirmatory laboratory testing.

**Case presentation:**

We analyzed genome sequences from a generally healthy population cohort of 2820 adults and identified PLG K330E in one individual. Subsequent review of this participant’s medical history revealed symptoms clinically attributed to allergy of unknown etiology but that are consistent with published descriptions of HAE patients carrying the PLG K330E variant. The participant, a 31 year old female, reported lip and tongue angioedema, without wheals, which did not respond to treatment with steroids or antihistamines.

**Conclusions:**

The genotype-first approach demonstrated that detection of PLG K330E in undiagnosed or misdiagnosed individuals can identify patients actually affected with HAE-U. The genetic diagnosis will facilitate selection of appropriate treatment, discontinuation of therapies ineffective for this condition, and timely diagnosis of affected family members. The results support a role of PLG K330E in the pathogenesis of HAE and suggest that genetic testing be considered as an approach to diagnose patients with unexplained angioedema.

## Background

Hereditary angioedema (HAE) is a potentially life-threatening set of disorders characterized by recurrent episodes of skin and submucosal edema, and occasionally intense abdominal pain. Because of the rarity of this group of disorders and the lack of specificity of the symptoms, the condition is difficult to diagnose. The resulting underdiagnosis or misdiagnosis leads to delays in recognizing patients affected with HAE and a lack of treatment or mismanagement of the disorder [1].

HAE has been classified into several types. Types I and II result from mutations in the gene *SERPING1*, which encodes C1 inhibitor (C1-INH), and HAE-FXII results from mutations in the gene *F12*, encoding coagulation factor XII. Types I and II may be diagnosed by laboratory testing for C1-INH deficiency, and HAE-FXII is confirmed by mutation analysis of *F12* exon 9. There is currently no established laboratory test to diagnose forms of HAE with unknown genetic cause (HAE-U), conditions with normal C1-INH and no detectable *F12* mutations.

Recently, two new genes have been proposed to underlie HAE-U, plasminogen (*PLG*) [2] and angiopoietin 1 (*ANGPT1*) [3]. These emerging associations are each based on a single variant, NM_000301.3:c.A988G (K330E) in *PLG* and NM_001146.4:c.G355T (A119S) in *ANGPT1*, with limited supporting data available to date. Both of these novel associations were unexpected. For *PLG*, previously reported mutations cause plasminogen deficiency type I and dysplasminogenemia, conditions without angioedema as a documented feature [4]. The *ANGPT1* association, based on segregation within a single family, expands the possible etiology of HAE from mutations affecting proteins of the plasma contact system to those of the vasculature [3].

The potential severity of HAE-U, compounded with the likelihood of underdiagnosis and misdiagnosis, makes it important to recognize this condition as early as possible. To investigate whether DNA sequencing should be considered for diagnosis of patients with unrecognized HAE-U, we analyzed genomic data from a cohort of individuals, unselected for HAE, for the PLG K330E and ANGPT1 A119S variants. Identification of PLG K330E in an individual with symptoms of unknown cause that are consistent with HAE-PLG supports both a role of this variant in the pathogenesis of HAE and also the use of genetic sequencing for diagnosis of patients with unexplained angioedema or non-confirmatory laboratory testing.

## Case presentation

Participants were enrolled in Inova Translational Medicine Institute’s “Childhood Longitudinal Health Outcomes” research study. The cohort is ancestrally diverse and includes 2820 adults, unselected for any genetic disorder [5]. DNA isolated from peripheral blood was sequenced to > 40× using Illumina whole genome sequencing technology as described [5]. Genomes were analyzed for the variants PLG K330E (hg19 chr6:161139762A>G), ANGPT1 A119S (chr8:108359268C>A), and also for SNPs and indels predicted to alter Thr328 in *F12* exon 9 (NM_000505.3). None of these *ANGPT1* and *F12* variants were found in the cohort, and PLG K330E was identified in one individual. The PLG K330E variant was confirmed by Sanger sequencing using a newly collected blood sample. No other variant in the four known HAE genes, *SERPING1*, *F12*, *PLG*, and *ANGPT1*, was found in this participant’s genome by searching for rare, predicted protein-impacting and promoter variants with the SAVANNA pipeline [6].

Subsequent review of the medical history of the individual carrying PLG K330E, of genomically-determined European ancestry, revealed that she is a 31 year old female who first began having lip and tongue angioedema at 21 years of age. Her episodes occur approximately once per year, only affect her lips and tongue, and present without wheals. The episodes usually begin in the morning when upon awakening she will notice mild swelling that gradually increases throughout the day. The onset of swelling is slow to develop, never rapid. The initial episodes involved only her lips; the last few episodes have involved her tongue as well. The swelling has always occurred unilaterally, with a very clear vertical line of demarcation along the midline of her lips or tongue. The swelling proceeds slowly, and typically only lasts 12 h. Her tongue swelling has never been severe enough to cause respiratory distress.

On several occasions, our patient sought out medical assistance for treatment, and was usually given Benadryl, steroids and an EPIPEN to carry. Believing the episodes were an allergic reaction, she visited an allergist, who helped her try to pinpoint an inciting factor, without success. The patient has taken oral contraceptives on and off since her late teenaged years. She has had three children, but did not note any increased frequency of symptoms during the pregnancies. The patient has one older sibling, and neither her sibling nor her parents have reported episodes of angioedema. Genomic data are unavailable for these family members.

Although HAE was not suspected prior to the genetic diagnosis, the symptoms of this individual are consistent with those of previously reported patients with a diagnosis or family history of HAE who carry the PLG K330E variant. These patients exhibit an increased tendency for edema to occur in the facial distribution, particularly in the tongue and lips, and a decreased tendency for the swelling to occur in the other parts of the body, and abdominal pain. Symptoms from HAE-PLG typically do not respond to steroids or antihistamines [2, 7–9]. Our patient has exclusively had swelling of the lips and tongue, and no episodes of abdominal pain. Her episodes were unprovoked, and resolved spontaneously.

## Discussion and conclusions

HAE is emerging as a diverse set of disorders that can be classified by the underlying causative mutations. Evolving understanding of the genetic basis of HAE with normal C1-INH has raised the possibility that HAE-U, in addition to HAE-FXII, may be diagnosed by mutation analysis. By applying a genotype-first approach to a population cohort of individuals unselected for HAE, we identified a participant carrying the PLG K330E variant who was subsequently found to have symptoms consistent with HAE-PLG that had been misattributed to allergy. This finding demonstrates that genetic analysis can provide a diagnosis for patients with angioedema of unknown cause, and supports the proposal that diagnostic testing include mutation analysis for PLG K330E [2].

Like other patients with HAE, the individual in our cohort was initially misdiagnosed and prescribed ineffective treatments. The genomics-driven diagnosis provided information critical for selecting appropriate therapy, based on reports of acute and prophylactic treatments that have been effective for other HAE-PLG patients, and for discontinuing treatments ineffective for this condition [2]. The genetic diagnosis will expedite assessment of affected family members and reduce delays in initiating appropriate management of the condition.

The physiological mechanism by which PLG K330E leads to angioedema is not yet known. Previous studies have suggested variable disease severity [2, 9], and asymptomatic carriers of this variant have been reported, suggesting incomplete penetrance [2]. We were unable to address the questions of variable expressivity and incomplete penetrance in this study because data from extended family members were unavailable.

The prevalence of HAE with normal C1-INH has been estimated at 1:100,000 [10], of HAE-U at 1:150,000 [10], and of HAE-PLG at about 1 in a million [11]. Although the patient reported here was identified by screening an unselected population, the rarity of the forms of HAE with normal C1-INH makes genetic screening of the general population for only these conditions impractical for routine diagnosis [2]. Rather, the pathogenic *PLG* and *ANGPT1* variants, in addition to variants in *F12*, could serve as diagnostic biomarkers for patients with unexplained angioedema or non-confirmatory laboratory testing, providing a molecular-level assay for establishing a diagnosis of HAE with normal C1-INH [12].

As genome-wide sequencing, including whole exome sequencing, becomes more widely incorporated into routine clinical care for all patients, detection of mutations in known HAE genes will likely identify additional patients not previously recognized as being affected with HAE. Even with the recent discoveries of associations of *PLG* and *ANGPT1* with HAE, it is expected that many patients with HAE-U will still have an unknown genetic basis, making it likely that additional causative HAE genes and variants will be found [12]. Identification of new genes and families with HAE with normal C1-INH will allow for studies investigating the mechanisms of pathogenesis of this set of disorders, and facilitate diagnosis of additional patients.

## Data Availability

The PLG variant was deposited in ClinVar with submission number SCV000844988.

## Abbreviations

C1-INH: C1 inhibitor
HAE: hereditary angioedema
HAE-U: hereditary angioedema with unknown genetic cause
SNPs: single nucleotide polymorphisms
